
JOURNAL INFORMATION
==============================
NLM Title Abbreviation: Wirtschaftsdienst
Journal ID: Wirtschaftsdienst
NLM Journal ID: 101086612

Wirtschaftsdienst (Hamburg, Germany : 1949)

ISSN: 0043-6275

ARTICLE INFORMATION
==============================
PMCID: PMC7678254
PMID: 33250531
DOI: 10.1007/s10273-020-2770-8
Article ID: 2770
Article version: 1
Subjects: Leitartikel

USA: Wahlsieg der Demokraten

Schwarzer Daniela schwarzer@dgap.org

grid.462094.d 0000 0001 0945 5851 Deutsche Gesellschaft für Auswärtige Politik e.V., Rauchstraße 17–18, 10787 Berlin, Deutschland
Electronic publication date: 2020 Nov 20
Print publication date: 2020
Volume: 100
Issue: 11
First page: 818
Last page: 819
Copyright: © Der/die Autor(en) 2020
License: Open Access: Dieser Artikel wird unter der Creative Commons Namensnennung 4.0 International Lizenz (https://creativecommons.org/licenses/by/4.0/deed.de) veröffentlicht.
Open Access wird durch die ZBW - Leibniz-Informationszentrum Wirtschaft gefördert.
License URL: https://creativecommons.org/licenses/by/4.0/

issue-copyright-statement: © The Author(s) 2020

==============================

Der Wahlsieg des Demokraten Joe Biden wurde in den meisten EU-Hauptstädten mit immenser Erleichterung aufgenommen. Der regierungserfahrene Transatlantiker, eine Art politisches Fossil, gilt als Garant eines belastbaren und berechenbaren Verhältnisses mit den USA. Biden wird als Partner gesehen, mit dem die regelbasierte internationale Ordnung wieder gestärkt und auch reformiert werden kann. Und doch wird es nie wieder so wie in guten alten transatlantischen Zeiten. Obwohl Biden mit deutlich mehr Stimmen gewählt wurde als jemals ein US-Präsident vor ihm, wird er mit inneren Widerständen und institutionellen Blockaden zu kämpfen haben. Die US-Wahl 2020 erzählt zwar die Geschichte einer historisch großen Mobilisierung von Wähler*innen. Doch wenn der Demokrat als 46. Präsident der Vereinigten Staaten von Amerika im Januar 2021 sein Amt übernimmt, wird er seine Energie zunächst nach innen richten müssen, um eine zutiefst gespaltene Gesellschaft wieder auf einende Ziele und ein Mindestmaß an Respekt und Solidarität auszurichten. Die Covid-19-Pandemie verschärft bestehende Ungleichheiten deutlich. Dieser soziale Sprengstoff fordert die amerikanische Demokratie heraus.

Am 4. Januar 2021 gibt es in Georgia eine Nachwahl für zwei Senatssitze. Geht nur einer davon an die Republikaner, haben sie in der Parlamentskammer eine knappe Mehrheit. Biden muss dann mit einer sogenannten geteilten Regierung arbeiten. Wenn das Weiße Haus und eine der beiden Kammern im Kongress von unterschiedlichen Parteien regiert werden, droht Stillstand, denn die Republikaner werden dem Demokraten an der Staatsspitze keinen politischen Erfolg schenken wollen und seine Vorhaben vereiteln oder zumindest verzögern. Bereits jetzt, im Nachgang zur Wahl, zeigt die Partei, dass sie keinen Schlussstrich unter die spaltende Brachialpräsidentschaft Donald Trumps zieht. Führende Republikaner bestreiten mit Trump den Wahlsieg Joe Bidens. Während Trump die Vorbereitung der Amtsübergabe blockiert, verbreiten viele von ihnen Verschwörungstheorien, nach denen Biden die Wahl „geklaut“ haben soll – aus Sorge, dass treue Trump-Wähler*innen an ihrer Basis bei der nächsten Wahl Rache nehmen könnten. So werden die Republikaner so lange wie möglich probieren, das nach Stimmen eindeutige Wahlergebnis politisch und juristisch anzufechten. Das ist nicht nur ein Bruch der Normen der US-amerikanischen politischen Kultur. Es zermürbt das Vertrauen in das politische System der USA.

Schon vor der Amtszeit Trumps konnte dort das politische System viele Probleme wegen innerer Blockaden nicht mehr lösen. Das steigerte die Politikverdrossenheit vieler Amerikaner*innen und verhalf dem Außenseiter Trump zum Wahlsieg. Er konnte dem politischen Establishment den Kampf ansagen und versprach der Bevölkerung, dass seine „Make America Great Again“-Politik auch im letzten Winkel der USA Vorteile haben würde. Je länger sich Covid-19 in den USA verbreitet, und je länger es dauert, einen Konsens über künftige Konjunkturmaßnahmen zu erzielen, desto später und zögerlicher kommt die wirtschaftliche Erholung. Ohne überparteiliche Einigung zur Verlängerung der Arbeitslosenhilfe verlieren Millionen von US-Haushalten die Finanzierung, die sie in der Corona-Krise über Wasser gehalten hat. Trump ist nicht die Ursache, sondern das Symptom tiefliegender Unzufriedenheiten und Spaltungen im Land, in dem sich Millionen entkoppelt fühlen, wirtschaftlich, sozial, geografisch und kulturell. 71 Mio. Wahlberechtigte haben bei der Präsidentschaftswahl erneut für ihn gestimmt. Sie könnten in vier Jahren einem ähnlich protektionistischen, diskriminierenden Kandidaten oder gar Trump selbst ein zweites Mal ins Amt verhelfen, sollte er nicht juristisch belangt werden. Es könnte sich im Rückblick herausstellen, dass nicht Trump die Ausnahme war, sondern dass dies Joe Biden ist.

Die innenpolitische Situation wird weitreichende Konsequenzen für die transatlantischen Beziehungen und für Europa haben. So wird die neue US-Regierung von Europa einen deutlich höheren Beitrag zur Lastenteilung im Rahmen der Nato einfordern. Insbesondere von Deutschland wird erwartet, dass die Verteidigungsausgaben und das tatsächliche Engagement steigen. Auch die Herangehensweise an die Handelspolitik wird von der angespannten wirtschaftlichen Lage bestimmt. Ein transatlantischer Handelskrieg dürfte vermieden werden. Aber die EU muss sich darauf einstellen, dass Biden konsequent US-amerikanische Wirtschaftsinteressen verfolgt. Auch er dürfte daher die deutschen und chinesischen Außenhandelsüberschüsse kritisieren. Maßnahmen zur Förderung des *Reshoring* wird der Demokrat vermutlich fortsetzen, um Arbeitsplätze zurück nach Hause zu holen und die Abhängigkeit etwa von China zu reduzieren. Es ist möglich, dass die „Buy America"-Regeln verschärft werden, auch zulasten europäischer Unternehmen. Ein umfassendes transatlantisches Handels- und Investitionsabkommen, wie einst TTIP, dürfte es in den kommenden Jahren nicht geben. Für die US-Regierung wird es aber wichtig sein, den Zugang zum europäischen Binnenmarkt, auch für Agrarprodukte, zu verbessern, denn auch viele Wähler*innen Bidens haben Sympathie für die Position, dass US-Interessen in der globalen Wirtschaft nur unzureichend geschützt sind. Zwei derzeit in Brüssel diskutierte Ideen dürfte die Administration derweil hart kritisieren: die Carbon Border Adjustment Measures im Handel und die angedachte europäische Besteuerung von digitalen Unternehmen.

Konstruktiv dürfte die transatlantische Kooperation in multilateralen Foren werden. Biden hat bereits angekündigt, dem Pariser Klimaschutzabkommen wieder beizutreten und dürfte auch die Weltgesundheitsorganisation (WHO) wieder stärken. In der Welthandelsorganisation (WTO) sollten sich die EU und die USA zunächst auf die Modernisierung der Regeln konzentrieren, etwa zu Industriesubventionen und zum digitalen Handel. In Bezug auf den Appellate Body der WTO dürfte Biden – anders als sein Vorgänger – offen sein für einen Reformdialog mit der EU. Das zentrale innen- und außenwirtschaftliche Thema der Biden-Regierung wird China sein. Trump hat die politische Aufmerksamkeit auf den Aufstieg Chinas und den relativen Machtverlust der USA gelenkt. Auch für die Biden-Administration dürfte Europa nicht mehr den zentralen Stellenwert haben, den es früher einmal hatte. Allerdings sind die Erwartungen groß: Die EU und ihre Mitglieder sollen sich im Ringen mit China klar an die Seite der USA stellen. Es wird eine wichtige transatlantische Diskussion, wie mit Nicht-Demokratien, insbesondere systemischen Wettbewerber*innen, international umgegangen wird. China ist eine technologiebasierte Autokratie mit internationaler Expansionsstrategie, missachtet Menschenrechte und kontrolliert seine Bevölkerung mit neuen Technologien. Deutschland und Europa haben demgegenüber – mit den USA – eine klare Positionierung, als westliche, liberale Demokratie. Allerdings haben viele EU-Staaten mit China ein enges wirtschaftliches Verhältnis mit gegenseitigen Abhängigkeiten, was die US-amerikanische *Decoupling*-Idee konterkariert. Auch mit Biden im Weißen Haus dürfte sich Europa gezwungen sehen, dieses Verhältnis weiter zu überprüfen, wie der Fall Huawei/5G zeigt. Darüber hinaus wäre ein strategischer Dialog zwischen den USA und der EU über den Umgang mit Autokratien in und außerhalb internationaler Organisationen ein wichtiger Schritt heraus aus der Trumpschen Ära. Die westliche Allianz kann im Systemwettbewerb mit China und Russland an Kraft zurückgewinnen – wenn politischer Wille und Durchsetzungskraft auf allen Seiten da sind.

Daniela Schwarzer
Deutsche Gesellschaft
für Auswärtige Politik
schwarzer@dgap.org

Daniela Schwarzer ist Direktorin der Deutschen Gesellschaft für Auswärtige Politik in Berlin.

